# Can apricot kernels fatty acids delay the atrophied hepatocytes from progression to fibrosis in dimethylnitrosamine (DMN)-induced liver injury in rats?

**DOI:** 10.1186/1476-511X-10-114

**Published:** 2011-07-07

**Authors:** Manal K Abdel-Rahman

**Affiliations:** 1 Nutrition and Food Science Department, Faculty of Home Economics, Helwan University, Cairo, Egypt

**Keywords:** Ground apricots kernel (GAK), Antioxidant activity, Cyanide, Dimethylnitrosamine, Liver fibrosis

## Abstract

**Background and aims:**

The present study was aimed to analyze the chemical composition of ground apricot kernel (GAK) and examine its effect on hepatic fibrosis *in vivo *induced by dimethylnitrosamine (DMN) in rats.

**Methods and results:**

Hepatic fibrosis was induced by intraperitoneal injections of 10 mg/kg DMN for 3 consecutive days each week over a period of 4 wk. The rats were randomly assigned to five groups of nine rats each: the negative control group (NC), the hepatic fibrosis group (PC), hepatic fibrosis supplemented with GAK (0.5 mg/kg/BW/rat), hepatic fibrosis supplemented with GAK (1 mg/kg/BW/rat) and hepatic fibrosis supplemented with GAK (1.5 mg/kg/BW/rat). Rats were killed, blood was collected and livers were excised for biochemical measurements and histological examination. Results indicate that the diet supplemented with GAK led to improving liver function, lipid peroxides, and liver CAT, SOD and GSH. These results were confirmed by liver histology. Hierarchically high levels f GAK (1.5 mg/kg/BW/rat) gave the best results compared to other tested levels.

**Conclusion:**

This study demonstrates that GAK administration specifically (1.5 mg/kg/BW/rat) can effectively improve liver fibrosis caused by DMN, and may be used as a therapeutic option and preventive measure against hepatic fibrosis. Furthermore, a human trial would be applied specially GAK is a part of Egyptian diet. The act of why high amounts of GAK was improved biochemical values compared to low or moderate levels tested in this study may be due to increase levels of oleic acid and other polyphenols in apricot kernels

## 1. Introduction

Apricot fruit is a part of Egyptian diet, is nutritionally contains carbohydrates, organic acids and mineral elements (iron, boron and potassium), vitamins such as pro-vitamin A, vitamins B, C and polyphenols [1,2]. Besides, phenolic compounds, such as quercetin, catechin, epicatechin, *p*-coumaric acid, caffeic acid, ferulic acid and their esters have been identified in the fruits [3]. Apricot kernel (AK) is traditionally roasting and mixing with coriander seeds and salt in a ground mixture called "Dokka" and eaten as part of Egyptian folk diet as a part of a meal or as a snack.

Apricot kernels are generally exported to European countries and used especially in medicine, cosmetic and oil production [4]. Durmaz and Alpaslan [5] mentioned that apricot kernels are added to bakery products (as whole kernels or ground) in retail bakeries and also consumed as appetizers.

Apricot kernels, particularly rich in lipid and protein, are potentially useful in human nutrition [6,7] along with significant amounts of oil and fiber [8,9]. In a previous study, reported that sweet apricot kernels contain more oil than bitter kernels, and that oleic acid and linoleic acid correspond to approximately 92 g/100 g of the total fatty acids present in apricot kernel Femenia et al. [6].

**Figure 1:**
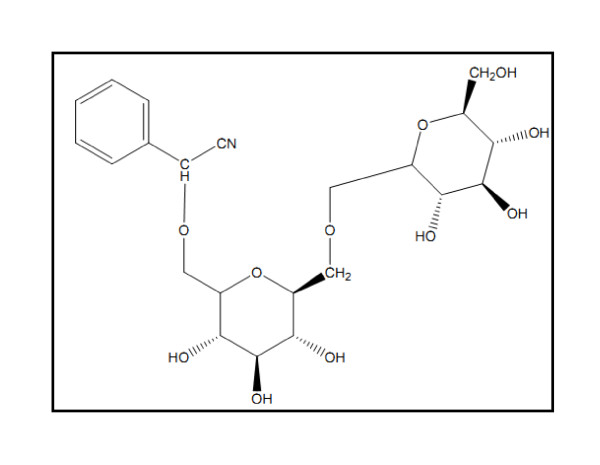
**The chemical structure of amygdalin**.

Amygdalin(Figure 1) is a major component of apricot kernels, bitter almonds and peach, plum, pear and apple seeds [10]. The amount of cyanogenic glycosides in plants varies with plant species and environmental effects [10]. For example, apricot seeds and bitter almonds contain approximately 20-80 μmol/g and 100 μmol/g of amygdalin, respectively.

Amygdalin (D-mandelonitrile-β-D-glucoside-6-β-glucoside) degrades to hydrogen cyanide, two molecules of glucose and benzaldehyde. Amygdalin hydrolysis is catalyzed by the enzyme emulsin, a β-glucosidase also found in apricot kernels. Since β-glucosidase enzymes do not occur intracellularly in humans, swallowing of whole apricot kernels may not release much cyanide [11], cyanogenetic glycosides may be essential for plant development and growth; the studies are represented by the work of [11]. However, the primary concern is their occurrence in food supplies and the potential adverse effects of long-term, low-level consumption. Cases of chronic cyanogen poisoning are documented in Third World areas, where cassava *(Manihot aesculenta) *that is a major food source [12].

After consumption of apricot and other fruit seeds, bitter almonds, cassava, or bamboo shoots, cyanide (CN) could be produced in high enough levels from the hydrolysis of amygdalin, and other cyanogenetic glycosides to pose a potential chronic toxicity problem [13,14]. These compounds may have evolved in plants as a chemical defense against grazing animals, as evidenced by the lethality of chokecherry leaves to livestock [15] and the apparent teratogenic effect of *P. armenlaca *in swine [16].

Liver injury is caused by different agents, such as viruses, chemicals, alcohol, and auto-immune diseases. It was pointed out that Dimethylnitrosamine (DMN) is a potent hepatotoxin that can cause fibrosis of the liver. At high doses, it is a "potent hepatotoxin that can cause fibrosis of the liver" in rats [17]. DMN is a potent hepatotoxin, carcinogen, and mutagen. DMN-induced liver injury in rats seems to be a good animal model for early liver cirrhosis [17]. A model of cirrhosis induced by chronic, discontinuous treatment with a low dose of DMN in the rat has been reported to reproduce a number of characteristics of this liver disease [18]. The extent of liver injury can be easily estimated by measuring the activities of certain plasma enzymes, e.g., alanine aminotransferase (ALT) and aspartate aminotransferase (AST).

Hepatic fibrosis is a common result of chronic injury to the liver [19]. Hepatic fibrosis is a consequence of severe liver damage and occurs in many forms of chronic liver damage, including virus infection, autoimmune liver diseases and sustained alcohol abuse [20]. However, the hepatoprotective effect of apricot kernel in dimethylnitrosamine (DMN)-induced models has rarely studied. The DMN-induced liver fibrosis model can reproduce most of the features observed during human liver fibrosis [21]. Furthermore, this model has other advantages such as progressive and remarkable pathological alterations, a high fibrosis reproduction rate, and a low mortality rate in experimental animals [22]. This model is also stable even after termination of DMN administration and is a reliable tool for screening antifibrotic agents [17]. Therefore, the aim of the present study was to examine the effect of ground apricot kernel (GAK) on hepatic fibrosis in rats.

## 2. Materials and methods

### 2.1. Materials

Dimethylnitrosamine (DMN) and all reagents and chemicals were purchased from El-Gomhorya Company, Cairo, Egypt.

Kits used to determine biochemical factors in serum were obtained from Egyptian American Company for Laboratory Service and Supplied by Alkan Company.

#### Apricot kernel

Apricots ***(Prunus armeniaca *L.) **was purchased from local fruit market (Giza-Egypt, June 2010). Apricot flesh was removed from fruits; the apricot outer shell was washed with tap water and air-dried at 30°C for about 2 weeks the outer shell of apricot was cracked manually and the edible part (kernel) was stored at -20°C in sealed plastic bags until used. The apricot kernels were soaked in warm distilled water for 1 h, kernel thin layer coat was removed manually. The apricot kernels were placed on a sheet of filter paper and dried under fume cupboard for 2 h and ground in a coffee machine for 1 min. The ground apricot kernels (GAK) were identified in the proximate analyses. In order to prevent the ground apricot kernel (GAK) from possible rancidity and oxidation that may occur during storage, the (GAK) was prepared freshly within 1 h before adding to basal diet.

#### Detoxification of apricot kernels

Detoxification of apricot kernels (AK) was conducted by soaking AK [23] in distilled water and ammonium hydroxide for 30 h at 47°C in order to decrease the total protein, non-protein nitrogen, total ash, glucose, sucrose, minerals, non-essential amino acids, polar amino acids, acidic amino acids, aromatic amino acids, anti-nutritional factors, hydrocyanic acid, tannins and phytic acid. On the other hand, remove toxic and bitter compounds from AK, increased the relative content of crude fiber, starch, and total essential amino acids.

### 2.2. Methods

#### Determination of total phenolics in GAK

A size of half gram of GAK were extracted in 50 mL of 62.5% aqueous methanol at 40°C for 2 h. Extracts were used promptly for analysis (in order to avoid any loss in polyphenols content as reported in [3]. Total phenolics were measured according to Waterman and Mole [24] using Folin-Ciocalteu assay. The Folin-Ciocalteu assay detects phenolics via oxidation of the phenol ion by a phosphotungstic-phosphomolybdic complex, resulting in blue coloration of the reduced chromophore. Fifteen microlitres of extract were added to 1·85 mL of de-ionized H_2_O and 75 μL of Folin-Ciocalteu reagent (Sigma Chemical Co., Cairo, Egypt), followed by adding 225 μL sodium carbonate after 5 min. After 2 h incubation at room temperature, absorbance of the solution at 760 nm was measured using a Helios Gamma Spectrophotometer (Unicam, Cambridge, UK), and compared with that of a condensed tannin standard (Sigma Chemical Co., Cairo, Egypt).

#### Determination of cyanide

Apricot kernels were obtained manually from apricot fruit and assayed for cyanide (CN) content by the method of Haque and Bradbury [25]. Amygdalin was determined as follows, 100 ml of 1 g HCN equivalents/l solutions were added to 0.1 M phosphoric acid and made up to 25 ml in a standard flask. Standard solutions of 1.00 g HCN equivalents/l of KCN and acetone cyanohydrin were prepared and added to 0.1 M phosphoric acid. To duplicate aliquots (2.00 ml) of these solutions was added 2.0 ml of 4 M sulphuric acid and the mixture heated for different times in a B14 stopper test tube in a boiling water bath, which just covered the liquid level in the test tube. Each sample was cooled in ice cold water, with the stopper loosely in place, 5.0 ml of 3.6 M sodium hydroxide was added and after 5 min, 1 ml was placed to 7 ml of 0.2 M acetate buffer at pH 5.0. Chloramine-T (0.4 ml), 5 min later 1.6 ml of isonicotinic acid/barbituric acid was added. After one hour, the absorbance was measured at 600 nm [26]. A calibration curve was obtained using a standard solution of KCN [26]. The amount of total cyanide present was obtained by linear extrapolation to zero time of the data. Ten replicate analyses were made in duplicate with amygdalin with linear extrapolation to zero time. Finely ground material (usually 100 mg) from GAK, was taken immediately after grinding and made up to 10.0 ml with 0.1 M phosphoric acid. The mixture was centrifuged and duplicate 2.00 ml taken for analysis as described above. The total cyanide content was obtained by linear extrapolation to zero time.

#### Proximate Analyses

The moisture content was determined by heating two grams of ground apricot kernel air-dried sample in a vacuum oven at 700°C under pressure of 95 mmHg to a constant weight [27]. The crude fat content was determined as follows: the oven-dried samples obtained from the moisture content determination was then extracted with petroleum ether (60-800°C) for 16 hours in Soxhlet-type extractor. The ether was evaporated and the residue dried to a constant weight at 95 - 1000°C and then cooled in a desiccator. The weight loss expressed as percentage gave the crude fat content [28].

The nitrogen percentage was determined by the improved Kjeldahl method described in AOAC [28] and the nitrogen content was converted to crude protein by multiplying with 6.25 [27]. The ceramic fiber filter method as described in AOAC [28] was used to analyze the crude fiber content. Briefly, two grams ground sample was defatted with petroleum ether then digested with 1.25% (v/v) H_2_SO_4 _and 1.25% (v/v) NaOH. The residues were ignited at 1300°C for 2 hours, cooled in a desiccator and weighed. The ash content was determined using AOAC recommended method [28]. The carbohydrate content of the sample was determined by subtracting the sum of the percentages of moisture, crude fat, fiber, protein and ash from 100.

#### Rats

Rats were purchased from the laboratory animal colony, Ministry of Health and Population, Helwan, Cairo, Egypt.

#### Experimental design

The animal experiment was conducted and approved according to the institutional guidelines of Ophthalmology Institute Guide Care of Animals (Giza-Egypt). Forty five males Sprague-Dawley rats weighing (200 ± 5 g) were kept in individual stainless steel cages under hygienic condition and were fed on basal diet for one week for adaptation according to Ain-93 [29]. After a period of adaptation, the rats were randomly assigned to five groups of nine rats each as follows:

**Group I: **fed on basal diet and used as a negative control group (NC).

**Group II: **injected with DMN and fed on basal diet, used as a positive control group (PC).

**Group III: **injected with DMN and fed on basal diet containing ground apricot kernel (GAK) in amount of 0.5 mg/kg body weight/rat.

**Group IV: **injected with DMN and fed on basal diet containing ground apricot kernel (GAK) in amount of 1.0 mg/kg body weight/rat.

**Group V: **injected with DMN and fed on basal diet containing ground apricot kernel (GAK) in amount of 1.5 mg/kg body weight/rat.

#### Inducing hepatic fibrosis in rats

Hepatic fibrosis was induced by intraperitoneal of dimethylnitrosamine (DMN) injected to the animals based on10 mg/kg for 3 consecutive days each week over a period of 4 wk according to Asakura et al, [30]. During the experimental period (4 weeks), diet consumed and body weights for rats were recorded twice a week and liver index, which is calculated as percent of liver weight at final body weight [31].

#### Biochemical analyses in the serum

At completion of the experiment, the animals were fasted overnight, anaesthetized with CO_2 _and sacrificed to obtain blood samples. Each blood sample was placed in dry clean centrifuge tube, and then centrifuged at 950 xg for 20 min. at 4°C to separate the serum. Serum was carefully separated into clean dry Wassermann tubes by using a Pasteur pipette and kept frozen at -20°C until analyses.

The liver was separated from each rat then washed thoroughly in ice-cold physiological saline [0.9% (w/v) NaCl], and weighed to calculate liver to body weight percentage.

Hepatotoxicity was assessed by quantifying the activities of serum alanine aminotransferase (ALT) and aspartate aminotransferase (AST), according to [32]. Superoxide dismutase (SOD) content was determined by the xanthine oxidase method.

#### Thiobarbituric acid-reactive substances (TBARS)

The content of serum lipid peroxides was analyzed by using thiobarbituric acid-reactive substances (TBARS) and expressing them as malonaldehyde equivalents using the method of Yagi [33] with reduced proportions. In brief, 20 μL serum was added to 2 mL 40 mmol/L H_2_SO_4 _(Sigma Pharmaceutical Industries, Cairo, Egypt), then 0.25 mL 10% wt/vol phosphotungstic acid (Sigma Chemical Co. Cairo, Egypt) was added and mixed. The mixture was centrifuged at 950 xg for 15 min., the supernatant was discarded, and the sediment was mixed with 1 mL 40 mmol/L H_2_SO_4 _and 0.15 mL 10% wt/vol phosphotungstic acid. The mixture was centrifuged at 950 xg for another 15 min. The sediment was suspended in 2 mL distilled water, and 0.5 mL 0.33% wt/vol thiobarbituric acid reagent (Sigma Pharmaceutical Industries, Egypt) was added. The mixture was heated for 60 minutes at 95°C in a water bath, then 2.5 mL *n*-butanol (Sigma Pharmaceutical Industries, Egypt) was added and the mixture was vigorously shaken. The butanol layer was taken after centrifugation at 950 xg for 15 minutes and absorbance was taken for fluorometric measurement at 553 nm with 515-nm excitation.

#### Biochemical analyses in liver tissues

Liver catalase (CAT) was determined by Goth's colorimetric method, in which supernatant was incubated in H_2_O_2 _substrate and the enzymatic reaction was stopped by the addition of ammonium molybdate. The intensity of the yellow complex formed by molybdate and H_2_O_2 _was measured at 405 nm [34].

Superoxide dismutase (SOD) activity was determined by using a measurement method developed by McCord and Fridovich [35]. This method is based on the generation of superoxide radicals produced by xanthenes and xanthenes oxidase, which react with 2-(4-iodophenyl)-3-(4-nitrophenol)-5-phenyltetrazolium chloride (INT) to form a red formazan dye. SOD activity was expressed as units per gram protein.

Glutathione peroxidase (GSH-Px) activity was measured on standard assay conditions in 340 nm (absorbance) at 37°C according to the method developed by Paglia and Valentine [36]. In this measurement, GSH-Px catalyzes the oxidation of glutathione by cumene hydroperoxide. Measurements were performed by an autoanalyzer (Biochemistry Dept., Faculty of Veterinary Laboratories, Cairo University) according to the Randox application procedure. GSH-Px activity was expressed as units per gram protein.

#### Histopathological studies

Specimens from left liver lobe were fixed in 10% formalin solution for 24 h, the fixed specimens were then trimmed, washed and dehydrated in bedded in paraffin, cut in sections of 46 microns thickness and stained with haematoxylin and eosin stain [37] and assessed in a light microscope (Nikon Eclipse E400). All alterations from the normal structure were registered. The following criteria were used for scoring liver histology; (0) indicates to no histopathological changes were observed and from 1-4 indicates to different degrees from histopathological changes that increase in numbers along with severity; for example 4 indicates to severe changes, 3 moderate, 2 mild and 1 slight changes.

#### Statistical analysis

The data analysis was carried out with SPSS Inc. software (version 15.0). One-way ANOVA was used to study a significant difference between means of the dietary groups with a significance level of P < 0.05. Duncan's test was used to compare the significance among the rat groups. All data are presented as ± standard deviation of means (STD) [38].

## 3. Results

The chemical composition of ground Apricot Kernels (GAK)

Results from (Table 1) indicate to the mean values of protein, carbohydrates, fiber, total polyphenols and cyanide in GAK.

**Table 1:** Analysis of ground apricot kernels (GAK)

Protein (g)	CHO (g)	Fiber (g) (Units/100 g)	Fat (g)	Total Phenolics (mg/g)	Cyanide (ppm)
16.04 ± 3.5	22.55 ± 6.5	4.29 ± 6.3	57.12 ± 2.4	2.5 ± 1.9	600

All values are the mean of 3 measurements, **(± STD)**

The distribution of fatty acids composition in GAK is given in (Table 2) The dietary GAK was rich in oleic (74.59%) acids, while linoleic acid represent (19.57%), with stearic, palmitic, palmitoleic and arachidic acids constituting 0.96%, 4.11%, 0.59%, and 0.18% respectively.

**Table 2:** GLC Fatty acids composition in GAK analysis%

OLEIC ACID (C18:1)	74.59%
Linoleic acid (C18:2)	19.57%

Stearic acid (C18:0)	0.96%

Palmitic acid (C16:0)	4.11%

Palmitoleic acid(C16:1)	0.59%

Arachidonic acid (C20:4)	0.18%

### Biochemical results

As shown in (Table 3) the liver index, (the percent of liver weight to final body weight), was significantly different among the experimental groups. Liver index was decreased in rat groups compared to negative control (NC).

**Table 3:** Relative organ weight in rats with DMN-induced hepatic fibrosis

Groups	Liver index
**I**	3.1 ± 0.5^d^

**II**	2.6 ± 0.4^a^

**III**	2.7 ± 0.3^b^

**IV**	2.9 ± 0.4^e^

**V**	3.0 ± 0.1^c^

Group I: fed on basal diet as a negative control group (NC). Group II: injected with DMN and fed on basal, used as a positive control group (PC). Group III: injected with DMN and fed on basal diet containing (ground apricot kernel)in amount of 0.5 mg/kg of body weigh/rat. Group IV: injected with DMN and fed on basal diet containing (ground apricot kernel) in amount of 1 mg/kg of body weigh/rat Group V: injected with DMN and fed on basal diet containing (ground apricot kernel) in amount of 1.5 mg/kg of body weigh/rat Values which don't share the same letter in each column are significantly different. Significance at P < 0.05, Liver index: Liver weight/body weight multiplied by 100.

AST and ALT concentrations in serum were used as biochemical markers to evaluate hepatic injury. ALT is a cytosolic enzyme, primarily present in the liver. An increase in serum ALT indicates liver damage more specifically than AST. AST, which is a mitochondrial enzyme present in large quantities in the heart, liver, skeletal muscle, and kidney, in part indicates liver injury. Serum activities of ALT and AST were found to be significantly increased in DMN group when compared with NC group (p < 0.05). A significant increase of the liver enzymes in the serum was occurred after dimethylnitrosamine (DMN) administration alone, which was significantly lowered by presence of GAK in III, IV and V rat groups (p < 0.05) (Table 4).

**Table 4:** Effect of different levels of GAK on liver functions in serum with DMN induced hepatic fibrosis in rats **(***n ***= **9)

Rat groups	AST (IU/mL)	ALT (IU/mL)	MDA (μmol)
**I**	85 ± 2.1^a^	243 ± 2.6^a^	2.30 ± 2.8^a^

**II**	451.2 ± 2.3^b^	582 ± 8.3^b^	2.97 ± 2.3^b^

**III**	261 ± 2.8^c^	446 ± 2.7^c^	2.48 ± 1.5^c^

**IV**	224 ± 2.4^d^	312 ± 2.9^d^	2.53 ± 3.1^c^

**V**	217 ± 4.7^d^	289 ± 4.1^d^	2.29 ± 2.1^a^

Group I: fed on basal diet as a negative control group (NC). Group II: injected with DMN and fed on basal, used as a positive control group (PC). Group III: injected with DMN and fed on basal diet containing (ground apricot kernel) in amount of 0.5 mg/kg of body weigh/rat. Group IV: injected with DMN and fed on basal diet containing (ground apricot kernel) in amount of 1 mg/kg of body weigh/rat Group V: injected with DMN and fed on basal diet containing (ground apricot kernel) in amount of 1.5 mg/kg of body weigh/rat Values which don't share the same letter in each column are significantly different. Significance at P < 0.05 (± SD).

**Figure 2:**
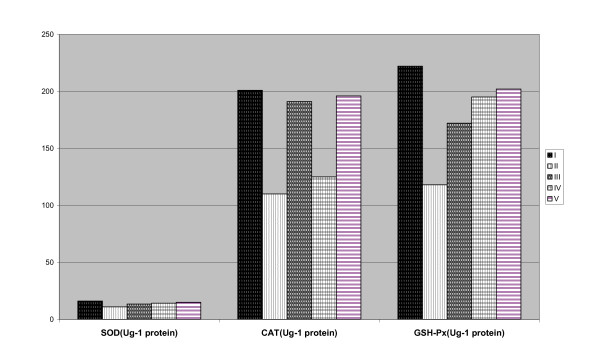
**Effect of feeding apricot kernels on SOD, CAT and GSH**. Group I: fed on basal diet as a negative control group (NC). Group II: injected with DMN and fed on basal, used as a positive control group (PC). Group III: injected with DMN and fed on basal diet containing (ground apricot kernel) in amount of 0.5 mg/kg of body weigh/rat. Group IV: injected with DMN and fed on basal diet containing (ground apricot kernel) in amount of 1 mg/kg of body weigh/rat Group V: injected with DMN and fed on basal diet containing (ground apricot kernel) in amount of 1.5 mg/kg of body weigh/rat Values which don't share the same letter in each column are significantly different. Significance at P < 0.05.

The value of MDA in the serum were decreased (p < 0.05) by the treatment of III, IV and V than that of the administration with DMN. The values of the activities of CAT and SOD in the liver tissue were increased (p < 0.05) with the addition of GAK among rat groups; III, IV and V compared to PC group (Figure 2).

### Histopathological results

**Figure 3:**
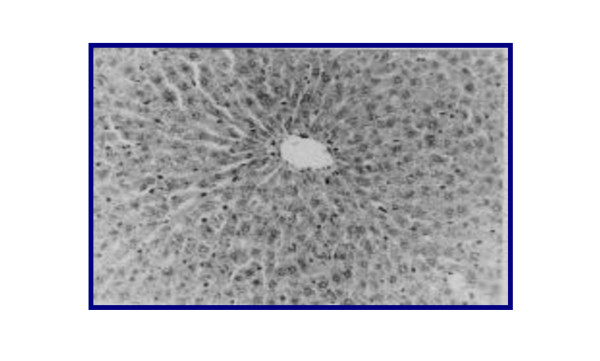
**liver of rats of control group showing no histopathological signs (Hand EX 200) (Grade 0)**.

**Figure 4:**
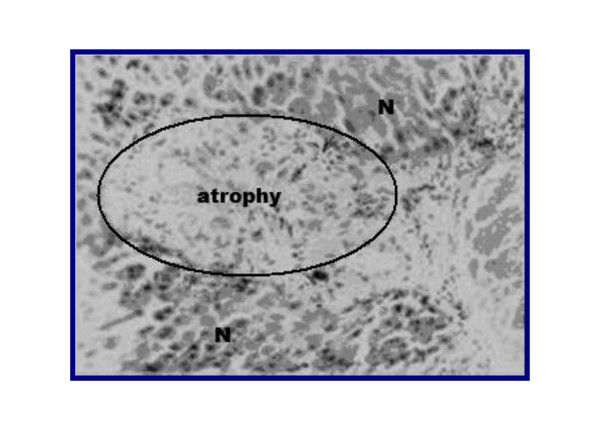
**liver of rat DMN-injected (II)**. The encircled area shows atrophied hepatocytes due to toxicity from DMN. The surrounding tissue looks normal (grade 4).

**Figure 5:**
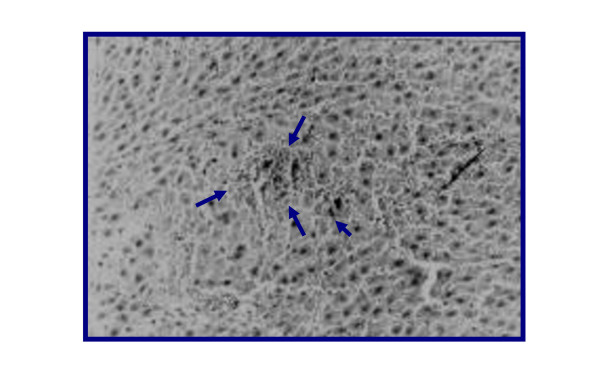
**(Another section) liver of rat DMN-injected (II) shows marked dilatation and congestion of hepatic simunsoids (small arrows) atrophy of hepatocytes and pylnosis of their nuclei (large arrows) (Grade 4)**.

Representative figures of hematoxylin and eosin-stained sections of liver tissue from NC group (I) (Figure 3) were evaluated as a normal histology. Effects of ground apricot kernel (GAK) on liver tissue morphology in DMN-induced fibrosis model was shown as follows; DMN-injected (PC) (Figure 4,5), DMN-injected + GAK (0.5 mg/kg/BW/rat). DMN-injected + GAK (1 mg/kg/BW/rat) and DMN-injected + GAK (1.5 mg/kg/BW/rat).

**Figure 6:**
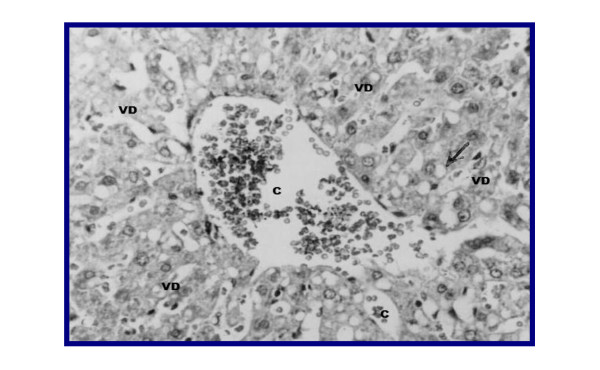
**liver of rat DMN-injected (III) +GAK (0.5 mg/kg/BW/rat)**. VD: vacuolar degeneration observed in hepatocytes. C: congested sinusoids and central vein (Grade 1.5).

**Figure 7:**
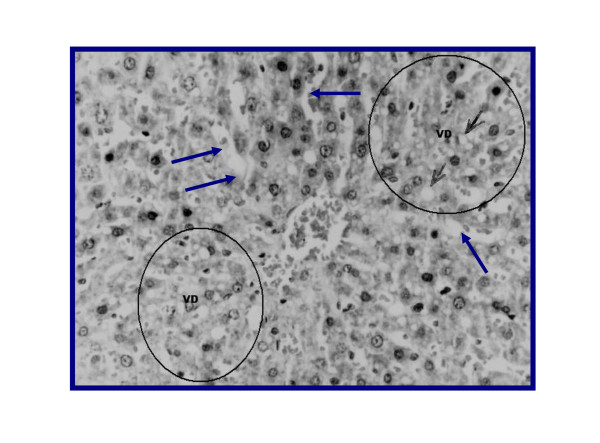
**liver of rat DMN-injected (III) + GAK (1 mg/kg/BW/rat)**. (Blue arrows): dilated and congested sinusoids, VD: hepatocytes showing vacuolar degeneration (Grade 2).

**Figure 8:**
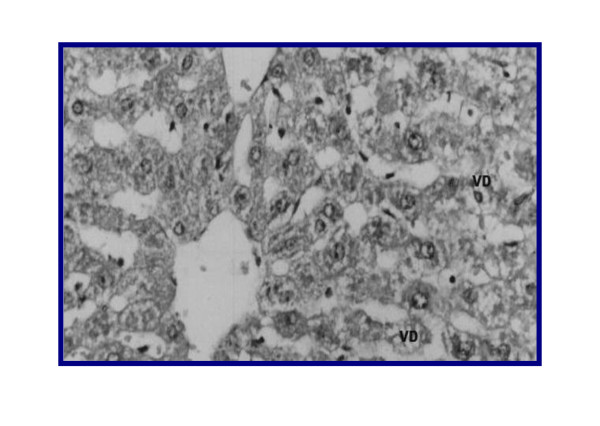
**liver of rat DMN-injected (IV) + GAK (1.5 mg/kg/BW/rat) (VD): Few vacuolar degenerated hepatocytes (Grade 0)**.

**Figure 9:**
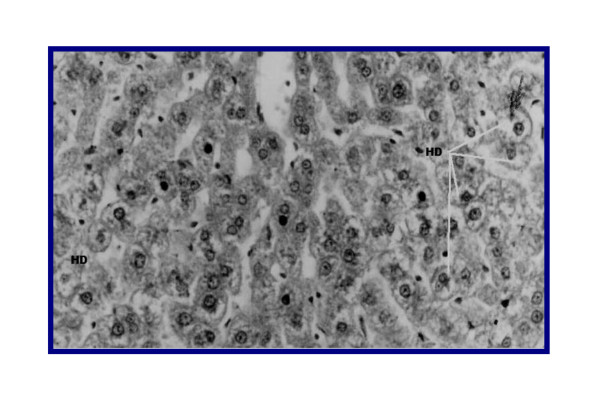
**(Another section) Liver of rat DMN-injected (IV) + AKG (1.5 mg/kg/BW/rat)**. Relatively normal hepatic architecture. (HD): Few hepatocytes demonstrate hydrotropic degeneration (Grade 0.5).

The NC showed normal architecture, whereas PC (II) (Figure 4, 5) showed atrophied hepatocytes, dilatation and congestion of hepatic sinusoids and atrophy of hepatocytes and pyknosis of their nuclei due to toxicity from DMN. Sections III (Figure 6), IV (Figure 7) and V (Figure 8, 9) showed a marked little decrease in the severity of hepatocellular necrosis compared to PC.

## 4. Discussions

In the present study, we focused our attention on the quantity of apricot kernel that showed an effective role in alleviating DMN-induced liver injury. For this purpose, GAK was separated into three levels in terms of amount added to the rats' diet and the effect of each level was investigated.

In this study, results showed that the treatment of rats' diet with ground apricot kernel (GAK) markedly inhibited acute hepatocellular injury in DMN model of liver toxicity. As reported previously, the initial step in liver injury caused by dimethylnitrosamine (DMN) is producing centrilobular necrosis of fairly rapid onset [39,40].

### Chemical analyses of GAK

The chemical composition of GAK indicates to high levels of oleic acid were found in GAK. It contains a substantial amount of dietary protein [41], along with significant amounts of oil and a fiber [8,9]. The result of the present study was in agreement with Montgomery [13] in the analysis of the chemical composition of GAK. In a study carried out by [13] demonstrates that dietary apricot kernel has preventive effects on *D*-galactosamine-induced liver injury. These results suggest that a prevention effect of liver injury that may be one of the important physiological property of dietary supplementation of the diet with apricot kernel. Similarly our study has revealed to the improvement effect of GAK in terms of biochemical and histological results.

It would be suggested that the chemical composition of ground kernel apricot has a role in these changes;[7,42] reported that oil content of kernels varies from 27.7 to 66.7%, majority of fatty acids being oleic (58.3-73.4%) and linoleic acids (18.8-31.7%). This component of monounsaturated fatty acid in apricot kernel was similar to olive oil content. That may be indicating the preventive role of apricot kernel from heart disease. Femenia et al [6] reported that sweet apricot kernels contain more oil than bitter kernels, and that oleic acid and linoleic acid correspond to approximately 92 g/100 g of the total fatty acids present. Similar results were found in the fatty acid composition of GAK.

The role of dietary antioxidants has also reported to protect from DMN toxicity; for example, potential physiologic activity of dietary flavonoids has often been the center of interest [43]. Phytosterols have been reported in measurable amounts in over 250 plants [44]. These compounds have a similar structure to cholesterol but with some modifications to the side chain, including the addition of a double bond and/or methyl or ethyl group. Interest in phytosterols has centered on their cholesterol lowering properties thereby offering protection from cardiovascular disease [45].

There are several reasons that may explain the potential effect of GAK to improve DMN liver toxicity; cultivars of apricot, presence of toxic cyanogenic glycoside amygdalin that alleviate the effect of DMN [46] or the nutritional value of GAK that analyzed in this study and other studies indicating to the presence of high amount of oleic acid and polyphenolics. Interestingly, Amygdalin can be hydrolyzed to form glucose, benzaldehyde and hydrocyanic acid also enzymatic release of cyanide occurs in the presence of β-glucoronidase, an enzyme found in the human intestine [47].

The antioxidant system involves both enzymatic and non-enzymatic agents. The first step in the enzymatic system is superoxide dismutase (SOD), which catalyzes the dismutation of superoxide anion (O·_2_) to H_2_O_2_. The conversion of H_2_O_2 _to H_2_O by either glutathione peroxidase (GPx) or catalase forms the second step of enzymatic system. Superoxide dismutase and GPx enzyme activities and the balance between them are very crucial for protection against oxidative stress [48,49]. The values of the activities of CAT and SOD in the liver tissue were enhanced by the presence of GAK for III, IV and V rat groups than that of the PC group (Figure 2).

Among all bimolecular, lipids are the most sensitive molecules to free radical attacks. Double bonds in fatty acids form peroxide products by reacting with free radicals, and products [namely malonaldehyde (MDA) can be formed in cell membranes]. Malonaldehyde shows both mutagenic and carcinogenic effects by changing membrane properties [50,51]. In our study, the results of malonaldehyde indicated that the supplementation of diet with different levels of GAK would alleviate the toxicity of DMN in rats. The value of MDA in the serum was decreased when supplementing rats' diets with GAK among III, IV and V rat groups compared to PC.

As a result of the degradation of lipid peroxides, MDA forms and is used as an indicator of lipid peroxidation [51]. Halliwel and Chirico [52] demonstrated the higher stability of saturated and monounsaturated oils in lipid peroxidation than that of polyunsaturated fatty acids because there is no conjugated sites in these fatty acids for oxidation. There are numerous harmful effects of MDA reported [53,54]. Cross linking with the membrane components, MDA causes inactivation of enzymes and receptors in membranes and thus changes membrane properties. Malondialdehyde also causes mutations by reacting with guanine nucleotide in DNA [55].

CAT is a hemoprotein, localized in the peroxisomes and catalyzes the decomposition of H_2_O_2 _to water and oxygen. GPx is a selenoenzyme, present predominantly in liver and catalyses the reaction of hydroperoxides with reduced glutathione to form glutathione disulphide (GSSG) and the reduction product of the hydroperoxide [56]. Increased activity of these antioxidant enzymes results in decreased formation of hydroxyl radical [57].

In the present study, the activity of SOD, CAT and GSH in the liver intoxicated with DMN (PC) - was significantly (*p  *< 0.05) decreased compared to NC group. The activities of these enzymes in the liver intoxicated with DMN - (III, IV and V groups) were significantly (*p *< 0.05) increased compared to PC group (Figure 2).

In this study, GAK administration prevented the development of hepatic fibrosis in a rat model of DMN-induced liver fibrosis at level of 1.5 mg/kg/BW/rat. These results were confirmed both by liver histology and biochemical analyses.

Because GAK can be consumed over long periods of time as traditional habits, in the present study apricot kernel possessed a therapeutic effect on DMN-induced hepatic fibrosis in rats through inhibiting liver inflammation and lipid peroxidation. Further studies are needed to confirm application of GAK on liver diseases in humans. The act of why high amounts of GAK was improved biochemical values compared to low or moderate levels that tested in this study; may be due to increase levels of oleic acid and other polyphenols in apricot kernels. Due to apricot kernels research warning from toxicity of apricot kernels due to presence of cyanide; this study detoxified apricot kernels and fed them to rats on different levels. We would suggest that the dietary form of apricot kernels mixture which called "Dokka" that is eaten in Egypt as a part of Egyptian dietary practices may have a detrimental health effect according to the toxicity levels. On the other hand, Dokka usually mixed with several herbs such as coriander, sesame and salt that may offset the side effect of toxicity. Because of high levels of salt in Dokka the dietary intake is not consumed in high amount which means that has a little detrimental effect on health.

## Competing interests

The author declares that they have no competing interests.

## Authors' contributions

The author conceived, designed and coordinated the work, as well as prepared the manuscript and carried out analytical work and statistical analysis and approved the final manuscript.
